# Cognitive Impairments in Alcohol-Dependent Subjects

**DOI:** 10.3389/fpsyt.2014.00078

**Published:** 2014-07-16

**Authors:** Florent Bernardin, Anne Maheut-Bosser, François Paille

**Affiliations:** ^1^Service d’Addictologie, CHU Nancy, Vandoeuvre, France; ^2^Faculté de Médecine, Université de Lorraine, Nancy, France

**Keywords:** alcohol-dependence, brain atrophy, cognitive impairments, inhibition, working memory, implicit cognition, cognitive remediation therapy

## Abstract

Chronic excessive alcohol consumption induces cognitive impairments mainly affecting executive functions, episodic memory, and visuospatial capacities related to multiple brain lesions. These cognitive impairments not only determine everyday management of these patients, but also impact on the efficacy of management and may compromise the abstinence prognosis. Maintenance of lasting abstinence is associated with cognitive recovery in these patients, but some impairments may persist and interfere with the good conduct and the efficacy of management. It therefore appears essential to clearly define neuropsychological management designed to identify and evaluate the type and severity of alcohol-related cognitive impairments. It is also essential to develop cognitive remediation therapy so that the patient can fully benefit from the management proposed in addiction medicine units.

Alcoholism causes a multitude of social and health problems with negative impact on quality of life and secondary costs to society (1–3). Evidence suggests that numerous determinants such as environmental, individual, and genetic factors could favor evolution toward alcohol-dependence. These factors may also interact with each other. Among environmental factors, quality of the neighborhood (4) or socio-economic factors (e.g., lower educational level, employment status) (5, 6) for example may increase risks of alcohol abuse. Individual and psychological characteristics including comorbid psychiatric disorders (7, 8), early life stress exposure (9), or impulsivity (10) are also risk-factors associated with chronic alcohol consumption. In addition, family, twin, and adoption studies have highlighted that genetic factors play an important role in the pathogenesis of alcohol-dependence (11–13). Heritability of alcohol-dependence is estimated between 50 and 80% (14) and is considered as a complex polygenic phenotype. In the same way, recent studies have examined cognitive endophenotype in alcoholism. They have shown that non-alcoholic relatives of alcohol-dependent individuals performed worse on cognitive tasks (specifically executive functions) and presented greater impulsiveness compared to control [e.g., Ref. (15)].

From a neurobiological perspective, alcohol-dependence is a chronic disorder, which implies the dopaminergic system. As seen in other drugs abuses, alcohol consumption acutely stimulates dopamine (DA) release from the major terminal area of the mesolimbic DA system, nucleus accumbens (NAC). Enhanced DA transmission in the NAC plays a critical role in the positive rewarding aspects of drugs abuses and the initiation of addictive process. Chronic administration is associated with functional alterations of this important part of the brain reward system. Globally, dysregulation of the dopaminergic system caused by chronic alcohol consumption produces drug dependence reinforcement and is most likely involved in the development of drug addiction (16–18).

The harmful effects of chronic alcohol consumption on the brain and cognitive functioning have been well described in the literature over recent decades (19). Cognitive impairments observed in alcohol-dependent patients not presenting any other neurological complications are increasingly becoming the focus of attention of addiction medicine professionals due to their impact on management, as, according to various studies, between 50 and 80% of these patients present impaired cognitive function (20, 21). These impairments are moderate to severe but usually remain undiagnosed when they are not specifically investigated. However, detailed neuropsychological assessment or screening of these cognitive impairments appears to be fundamental to optimally adapt patient management strategies.

## Alcohol-Related Cognitive Impairments

Neuroanatomical alterations can account for cognitive impairments affecting various functions, primarily executive functions (22). Fifty to 80% of patients present alterations of cognitive functions that probably impact on their management. However, there is a marked interindividual variability of the nature and severity of these impairments.

Ihara et al. (23) defined four profiles in these patients: (1) no cognitive impairment, (2) isolated executive deficits with no impairment of memory and global cognitive efficiency, (3) modified dysexecutive syndrome with memory impairments and preservation of global cognitive efficiency, and (4) global impairment (executive, memory, and global cognitive efficiency). More specifically, the dysexecutive syndrome can affect various processes such as working memory (24), mental flexibility (25), divided attention (26), decision-making (27, 28), or problem-solving (29). Disorders of prepotent response inhibition play a predominant role in addiction medicine to the point that alcohol-dependence has been described as a “disinhibitory disorder” (28).

The characteristic profile of alteration of episodic memory in alcohol-dependent patients comprises limited learning capacities, impairments of encoding, and recollection processes, difficulties recalling the temporospatial context and deficits of autonoetic consciousness, while information storage is preserved (25, 30). Alteration of executive functions, particularly disorders of inhibition, flexibility, or dual-task coordination also constitute predictive factors of memory impairment (25, 30). In contrast, apart from obvious deficits (i.e., related to dysexecutive syndrome), there is also probably a genuine impairment of episodic memory likely due to the hippocampal atrophy observed in these patients (25).

Finally, visuospatial functions are also predominantly affected, as several studies have demonstrated impaired performances on visuospatial processing, memory and visual learning, visuospatial organization, and visuoconstruction tasks (31, 32).

Three main hypotheses have been proposed in the literature to account for the characteristic cognitive profile observed in alcohol-dependent patients [see Ref. (31) for a detailed review of these hypotheses].

The first hypothesis is based on the pervasiveness and impaired recovery after withdrawal of visuospatial cognitive functions that are attributed to the non-dominant hemisphere. The right hemisphere would therefore be more susceptible to the neurotoxic effects of alcohol (32). However, this postulate has been questioned by contradictory results (31). The second hypothesis proposes that the increased susceptibility of frontal structures would account for the cognitive profile dominated by executive deficits (33). However, this hypothesis also appears to be too restrictive, as other cerebral structures are also involved (34). The third hypothesis, based on neuroanatomical and neuropsychological data, reconciles the previous two hypotheses by postulating the existence of global brain damage (35). In their meta-analysis of neuroanatomical data, the authors emphasized not only the existence of right hemisphere and frontal lobe lesions, but also lesions affecting other cerebral regions (medial temporal, subcortical, and cerebellar atrophy). The neuropsychological functional deficits observed in these patients also concern several cognitive functions in both verbal and visual modalities, which constitutes an additional argument in favor of the global brain damage hypothesis.

## Anatomical Lesions

Chronic excessive alcohol consumption induces global brain atrophy characterized by reduction of brain volume and enlargement of the ventricles and sulci (36). The severity of brain damage depends on various factors such as the extent of alcohol consumption, age, gender, and neurological or psychiatric comorbidities (31). The most susceptible brain structures are the neocortex in the frontal lobes, the limbic system, and the cerebellum (37). Reduction of gray matter preferentially involves frontoparietal regions, while reduction of white matter tends to be more diffuse (22).

More precisely, Kril et al. (38) estimated that chronic alcohol consumption induced about 15–23% loss of neuronal density in the frontal regions. This result is concordant with those of a more recent study, which demonstrated a 20% reduction of the gray matter volume in the dorsolateral prefrontal cortex (22). Pfefferbaum et al. (39) showed that aging brain is more sensitive to the deleterious effects of excessive alcohol consumption. This study indicates that the profile of brain damage in the frontal lobes varies according to the patient’s age even after accounting for the effects of normal aging and regardless of amounts of lifetime consumption of alcohol or duration of illness. Therefore, these results indicate an increased susceptibility of the brain in the elderly according to the model of alcohol-related premature aging of the brain (40). Furthermore, many studies have also demonstrated functional changes with a reduction of glucose metabolism or cerebral blood flow in prefrontal regions, particularly in the medial temporal region (37).

Alcohol-related brain damage also concerns the limbic system and particularly the hippocampus, regions involved in episodic memory (41–44). The hypothalamus and mammillary bodies are also particularly susceptible to chronic excessive alcohol consumption, especially when it is accompanied by vitamin deficiencies as in Wernicke’s encephalopathy or Korsakoff’s syndrome.

Finally, the cerebellum is also affected in these patients, with a reduction of the white matter volume in the vermis and cerebellar hemispheres (40). A study of the connections between the cerebellum and frontal regions via the pons and thalamus also demonstrated alteration of the frontocerebellar circuit (34).

## Detection and Diagnosis

In this context, the detection of cognitive impairments in alcohol-dependent patients is therefore essential and should be systematic. The Montreal Cognitive Assessment (MoCA) Test appears to be the most appropriate screening test for detection of cognitive impairments in these patients (45), as this tool is more sensitive than the Mini Mental State Examination (MMSE) for mild-to-moderate cognitive impairments (46). The MoCA Test can be performed by medical students, medical doctors, or certified neuropsychologist. Detection of cognitive impairments can then lead to referral of the patient for neuropsychological diagnostic assessment performed by a clinical neuropsychologist. Neuropsychological assessments can last 2–3 h and are designed to demonstrate preservation or impairment of the most susceptible cognitive functions in this population. Clinical neuropsychologists have at their disposal a battery of tests to evaluate several cognitive domains such as memory (e.g., California Verbal Learning test and Doors and People test), executive functions (e.g., Trail Making Test part B and Wisconsin Card Sorting Test, the Stroop Color Word test, and the Letter Fluency Test), working memory (Digit Span and Letter-Number Sequencing test), or processing speed (Digit Symbol-Coding).

In the alcohol-dependent population, the most susceptible executive processes to be evaluated are working memory, mental flexibility, inhibition, processing speed, concept formation, planning, and problem-solving capacities. Evaluation of verbal and visual memory must focus on encoding, recall, storage, learning, and recognition capacities, while assessment of visuospatial functions must focus on visuospatial organization and visuoconstruction capacities.

## Alcohol Consumption and Implicit Cognitive Processes

The study of the implicit mechanisms involved in addictive behavior has been considerably developed over recent years. The addictive problem of alcohol-dependence results from a conflict between an urge to drink and the desire to limit alcohol intake. Dual-process models can explain this conflict by the fact that drinking behavior involves two cognitive systems: an impulsive system and a reflexive system (47). The impulsive system is a “bottom-up” system, based on implicit cognitive processes that play a role in automatic behavior via strong associative memory between alcohol-related cues, outcome, and behaviors. This system therefore reinforces the incentive effects between stimuli related to the addiction (odors, places of consumption, or advertisements related to alcohol, for example) and the addictive behavior. It mobilizes the dopaminergic system of the amygdala–striatal circuit. The reflective system is a “top-down” system based on controlled cognitive processes, in which executive functions regulate the impulsive system to ensure adapted behavior. The underlying network involves various regions of the frontal lobe (lateral inferior prefrontal, dorsolateral, ventromedial, orbitofrontal, and frontoparietal) and the striatum. Finally, the insula plays a decisive role in the articulation of these two systems by translating unconscious interoceptive signals (or somatic states) into conscious subjective experiences (desires or needs) involved in the decision-making process. This system would therefore play a conflict management role between a stimulus related to addiction and a potentially associated somatic state (for example withdrawal symptoms) in order to guide decision-making.

The interaction between the two systems has been clearly documented in the field of alcohol-dependence (48). According to this theory, drinking behavior is activated by automatic processes (impulsive system) unless the subject is able to ensure control by mobilizing executive functions (reflective system). The impairment of executive functions observed in alcohol-dependent patients would therefore predispose to drinking behavior dictated by the impulsive system. More precisely, disorders of inhibition capacities and working memory play a predominant role in this dysregulation of the impulsive system by the reflective system (28, 47, 49–54), thereby resulting in a vicious circle, as chronic excessive alcohol consumption induces working memory and inhibition disorders that are then responsible for dysfunction of the reflective system. Finally, alcohol consumption results exclusively from mobilization of the impulsive system that perpetuates the addictive behavior resulting in continuing deterioration of executive functions (53).

## Cognitive and Brain Recovery

The study of alcohol-dependent patients also constitutes a model of brain plasticity, as an increase of brain volume characterized by increased white matter and gray matter volumes and a reduction of the size of sulci and ventricles is observed right from the first months of abstinence (55–60). The cognitive effects of this recovery consist of improvement of executive functions and verbal episodic memory (21, 58, 60–65).

In parallel, it has been shown that new brain regions can be recruited by recently weaned alcohol-dependent patients to compensate for alcohol-related brain damage (66, 67). Neuroadaptation mechanisms therefore enable patients to maintain a similar level of performance on cognitive tasks to that of control subjects. For example, alcohol-dependent patients recruit neuronal networks parallel to the frontocerebellar circuit normally used by control subjects to perform executive tasks (68).

However, although abstinence allows an improvement of cognitive functions, this is only achieved after a period of several months. A recent meta-analysis showed that, despite studies showing early cognitive recovery, a global deficit was still present several months after installation of abstinence and the cognitive profile tended to become normal only after 1 year of abstinence, while certain residual cognitive impairments may persist. For example, the presence of visuospatial function deficits may be observed after several years of abstinence, related to the decreased volume of the right parietal cortex (35).

## Cognitive Impairments and Management of Alcohol Abuse

Appropriate management of alcohol withdrawal is mandatory to prevent severe complications like delirium tremens or epileptic seizure. Prevention of Wernicke’s encephalopathy relies on thiamine prescription. If benzodiazepines usage and appropriate rehydration are codified, the dose and duration of thiamine treatment remains unclear (69). Nevertheless long-term abstinence is the main goal and cognitive behavioral therapy (CBT) and psychosocial programs are necessary. Adjuvant treatments may include: glutamate antagonist (acamprosate) or opioid antagonist (naltrexone).

The presence of cognitive impairments therefore requires adaptation of the management of alcohol-dependent patients. CBT has been demonstrated to be effective in the management of alcohol-dependence (70), but it is somewhat paradoxical to propose management that directly involves cerebral structures and cognitive functions altered by chronic alcohol consumption. This management approach may therefore be inappropriate or at least insufficient for a certain number of patients.

The efficacy of CBT would therefore depend on the integrity of certain brain regions of interest. For example, it has been shown in schizophrenic patients that the volume of gray matter in the frontal, temporal (including hippocampus), parietal, and cerebellar regions, brain regions that are also damaged in alcohol-dependent patients, is predictive of the efficacy of management (71). Similarly, the integrity of the frontocerebellar network, a site of predilection for brain damage in alcohol-dependent patients, would play an essential role in the efficacy of CBT due to its role in executive functioning (72).

Cognitive behavioral therapy in addiction medicine also requires elaborate cognitive capacities such as episodic, semantic and procedural memories, and executive functions (23, 73–77). This type of management could therefore be unsuitable for patients with cognitive impairments (25, 77–79). Various studies have shown that alcohol-dependent patients with the most severe cognitive impairment also have the least favorable prognosis (80–82).

Cognitive impairment can also influence the expression of individual and environmental factors involved in management, such as self-efficacy, readiness to change, active participation in group therapy, or treatment compliance, as the initial cognitive impairment is predictive of poorer treatment compliance and a decreased self-efficacy. Patients with severe cognitive impairments are also less able to use their own resources during management, in which case the prognosis depends more on the role of external factors such as group therapy or the family support network (83).

Finally, Le Berre et al. (80) demonstrated the role of cognitive functions in the motivation process of patients to change their addictive behavior. In their study, the authors used the motivational model described by Prochaska and DiClemente (84), which defines three stages of change as the key to the patient’s commitment to the management process: the precontemplation stage (the subject has no intention to change his/her behavior), the contemplation stage (the subject considers changing his/her behavior but remains ambivalent), and the action stage (cessation of consumption and setting-up of strategies to change behavior). Based on this model, the authors showed that episodic memory plays a role in the subject’s awareness of the addictive behavior and the need for follow-up. The integrity of this function actually determines the subject’s passage from the precontemplation stage to the contemplation stage. Similarly, the integrity of executive functions enables patients to weigh up their decisions to reach the action stage, which can only be implemented when decision-making capacities are preserved. Cognitive impairment therefore influences the degree of motivation of alcohol-dependent patients, an essential prerequisite to the success of management.

The presence of cognitive impairments on admission and during the first months of abstinence therefore influences management at various levels by determining the efficacy of treatment and the prognosis for lasting abstinence. Addiction medicine management must therefore be adapted to alcohol-dependent patients with cognitive impairments.

## Cognitive Remediation Therapy

In the light of these findings, it appears essential to propose management based on programs ranging from cognitive remediation to optimal use of the remaining capacities. However, very few addiction medicine units propose cognitive remediation therapy and very few studies have investigated this problem in alcohol-dependent patients. The majority of studies in the field are now relatively old (77, 79) and no longer correspond to current methodological requirements. However, the results of studies conducted in this field are encouraging. A recent study demonstrated that a cognitive remediation program was able to improve divided attention, alert capacities, working memory, and episodic memory. In addition to cognitive improvement, cognitive remediation therapy also improves other non-cognitive domains, especially psychological aspects (well-being, self-esteem) and craving (85).

Studies of the implicit mechanisms involved in addictive behavior also constitute a field of predilection for cognitive remediation. A series of such studies has shown that training working memory and inhibition can lessen the impact of these implicit process on drinking behavior (49, 51, 52, 86, 87). However, the methodology of these studies has been the subject of criticism (87) and the observed modification of drinking behavior also does not appear to be related to improvement of inhibition capacities but rather to the formation of new implicit associations promoting an impulsive drinking avoidance response (50). Finally, only one of these studies has proposed a cognitive remediation therapy that can be transposed to clinical practice (52) and that complies with the clinical criteria defined in the field of cognitive remediation (88). The results of these studies therefore need to be confirmed by further study protocols satisfying the methodological requirements in the field of cognitive remediation therapy in neuropsychologia.

## Conclusion

Data published in the literature suggest that it is essential to take into account the cognitive dimension of alcohol-dependent patients in order to adapt their treatment and to palliate their difficulties in activities of daily living. The brain changes and the profile of cognitive impairments presented by patients with chronic excessive alcohol consumption have now been very extensively documented in the literature. The role of these changes on drinking behavior, especially via the cognitive processes involved in the mechanisms of addiction, also constitutes a rapidly growing new field of research. Finally, the impact of these impairments on the modalities and efficacy of the proposed management is a clinical problem systematically raised in research. The last domain to be developed in the field of management of alcohol-dependent patients is therefore that of cognitive remediation, which can establish the link between the various problems related to cognitive deficits in the clinical management of these patients in order to propose specific targeted follow-up in a remediation therapy program devoted to these impairments.

## Conflict of Interest Statement

The authors declare that the research was conducted in the absence of any commercial or financial relationships that could be construed as a potential conflict of interest.

## References

[1] Laramée P Kusel J Leonard S Aubin H-J François C Daeppen J-B The economic burden of alcohol dependence in Europe. Alcohol Alcohol (2013) 48(3):259–69 10.1093/alcalc/agt004 23371284

[2] Rehm J The risks associated with alcohol use and alcoholism. Alcohol Res Health (2011) 34(2):135–43 22330211 PMC3307043

[3] Whiteford HA Degenhardt L Rehm J Baxter AJ Ferrari AJ Erskine HE Global burden of disease attributable to mental and substance use disorders: findings from the Global Burden of Disease Study 2010. Lancet (2013) 382(9904):1575–86 10.1016/S0140-6736(13)61611-6 23993280

[4] Bernstein KT Galea S Ahern J Tracy M Vlahov D The built environment and alcohol consumption in urban neighborhoods. Drug Alcohol Depend (2007) 91(2–3):244–52 10.1016/j.drugalcdep.2007.06.006 17644274

[5] Galvan FH Caetano R Alcohol use and related problems among ethnic minorities in the United States. Alcohol Res Health (2003) 27(1):87–94 15301403 PMC6676695

[6] Bryden A Roberts B Petticrew M McKee M A systematic review of the influence of community level social factors on alcohol use. Health Place (2013) 21:70–85 10.1016/j.healthplace.2013.01.012 23454663

[7] Dawson DA Grant BF Stinson FS Chou PS Psychopathology associated with drinking and alcohol use disorders in the college and general adult populations. Drug Alcohol Depend (2005) 77(2):139–50 10.1016/j.drugalcdep.2004.07.012 15664715

[8] Grant BF Hasin DS Stinson FS Dawson DA June Ruan W Goldstein RB Prevalence, correlates, co-morbidity, and comparative disability of DSM-IV generalized anxiety disorder in the USA: results from the National Epidemiologic Survey on Alcohol and Related Conditions. Psychol Med (2005) 35(12):1747–59 10.1017/S0033291705006069 16202187

[9] Enoch M-A The role of early life stress as a predictor for alcohol and drug dependence. Psychopharmacology (2011) 214(1):17–31 10.1007/s00213-010-1916-6 20596857 PMC3005022

[10] Jentsch JD Ashenhurst JR Cervantes MC Groman SM James AS Pennington ZT Dissecting impulsivity and its relationships to drug addictions. Ann N Y Acad Sci (2014). 10.1111/nyas.12388 24654857 PMC4360991

[11] Wetherill L Agrawal A Kapoor M Bertelsen S Bierut LJ Brooks A Association of substance dependence phenotypes in the COGA sample. Addict Biol (2014). 10.1111/adb.12153 PMC4233207 24832863

[12] Schuckit MA An overview of genetic influences in alcoholism. J Subst Abuse Treat (2009) 36(1):S5–14 19062348

[13] Kimura M Higuchi S Genetics of alcohol dependence. Psychiatry Clin Neurosci (2011) 65(3):213–25 10.1111/j.1440-1819.2011.02190.x 21507127

[14] Knopik VS Heath AC Madden PAF Bucholz KK Slutske WS Nelson EC Genetic effects on alcohol dependence risk: re-evaluating the importance of psychiatric and other heritable risk factors. Psychol Med (2004) 34(8):1519–30 10.1017/S0033291704002922 15724882

[15] Gierski F Hubsch B Stefaniak N Benzerouk F Cuervo-Lombard C Bera-Potelle C Executive functions in adult offspring of alcohol-dependent probands: toward a cognitive endophenotype? Alcohol Clin Exp Res (2013) 37(Suppl 1):E356–63 10.1111/j.1530-0277.2012.01903.x 23240659

[16] Gonzales RA Job MO Doyon WM The role of mesolimbic dopamine in the development and maintenance of ethanol reinforcement. Pharmacol Ther (2004) 103(2):121–46 10.1016/j.pharmthera.2004.06.002 15369680

[17] Pierce RC Kumaresan V The mesolimbic dopamine system: the final common pathway for the reinforcing effect of drugs of abuse? Neurosci Biobehav Rev (2006) 30(2):215–38 10.1016/j.neubiorev.2005.04.016 16099045

[18] Söderpalm B Ericson M Neurocircuitry involved in the development of alcohol addiction: the dopamine system and its access points. Curr Top Behav Neurosci (2013) 13:127–61 10.1007/7854_2011_170 22094880

[19] Fitzhugh LC Fitzhugh KB Reitan RM Adaptive abilities and intellectual functioning in hospitalized alcoholics. Q J Stud Alcohol (1960) 21:414–23 13700068

[20] Fein G Bachman L Fisher S Davenport L Cognitive impairments in abstinent alcoholics. West J Med (1990) 152:531–7 2190421 PMC1002406

[21] Bates ME Voelbel GT Buckman JF Labouvie EW Barry D Short-term neuropsychological recovery in clients with substance use disorders. Alcohol Clin Exp Res (2005) 29:367–77 10.1097/01.ALC.0000156131.88125.2A 15770112 PMC3059764

[22] Chanraud S Martelli C Delain F Kostogianni N Douaud G Aubin H-J Brain morphometry and cognitive performance in detoxified alcohol-dependents with preserved psychosocial functioning. Neuropsychopharmacology (2007) 32:429–38 10.1038/sj.npp.1301219 17047671

[23] Ihara H Berrios GE London M Group and case study of the dysexecutive syndrome in alcoholism without amnesia. J Neurol Neurosurg Psychiatry (2000) 68:731–7 10.1136/jnnp.68.6.731 10811696 PMC1736975

[24] Noël X Paternot J Van der Linden M Sferrazza R Verhas M Hanak C Correlation between inhibition, working memory and delimited frontal area blood flow measure by 99mTc-Bicisate SPECT in alcohol-dependent patients. Alcohol Alcohol (2001) 36:556–63 10.1093/alcalc/36.6.556 11704622

[25] Pitel AL Beaunieux H Witkowski T Vabret F Guillery-Girard B Quinette P Genuine episodic memory deficits and executive dysfunctions in alcoholic subjects early in abstinence. Alcohol Clin Exp Res (2007) 31:1169–78 10.1111/j.1530-0277.2007.00418.x 17511749 PMC2895973

[26] Tedstone D Coyle K Cognitive impairments in sober alcoholics: performance on selective and divided attention tasks. Drug Alcohol Depend (2004) 75:277–86 10.1016/j.drugalcdep.2004.03.005 15283949

[27] Le Berre A-P Rauchs G La Joie R Mézenge F Boudehent C Vabret F Impaired decision-making and brain shrinkage in alcoholism. Eur Psychiatry (2012) 29:125–33 10.1016/j.eurpsy.2012.10.002 23182846

[28] Noël X Van der Linden M d’ Acremont M Bechara A Dan B Hanak C Alcohol cues increase cognitive impulsivity in individuals with alcoholism. Psychopharmacology (2007) 192:291–8 10.1007/s00213-006-0695-6 17279375

[29] Noël X Van der Linden M Schmidt N Sferrazza R Hanak C Le Bon O Supervisory attentional system in nonamnesic alcoholic men. Arch Gen Psychiatry (2001) 58:1152–8 10.1001/archpsyc.58.12.1152 11735844

[30] Noël X Van der Linden M Brevers D Campanella S Hanak C Kornreich C The contribution of executive functions deficits to impaired episodic memory in individuals with alcoholism. Psychiatry Res (2012) 198:116–22 10.1016/j.psychres.2011.10.007 22377577

[31] Oscar-Berman M Marinkovic K Alcohol: effects on neurobehavioral functions and the brain. Neuropsychol Rev (2007) 17:239–57 10.1007/s11065-007-9038-6 17874302 PMC4040959

[32] Ratti MT Bo P Giardini A Soragna D Chronic alcoholism and the frontal lobe: which executive functions are imparied? Acta Neurol Scand (2002) 105:276–81 10.1034/j.1600-0404.2002.0o315.x 11939939

[33] Uekermann J Daum I Schlebusch P Wiebel B Trenckmann U Depression and cognitive functioning in alcoholism. Addiction (2003) 98:1521–9 10.1046/j.1360-0443.2003.00526.x 14616178

[34] Sullivan EV Compromised pontocerebellar and cerebellothalamocortical systems: speculations on their contributions to cognitive and motor impairment in nonamnesic alcoholism. Alcohol Clin Exp Res (2003) 27:1409–19 10.1097/01.ALC.0000085586.91726.46 14506401

[35] Stavro K Pelletier J Potvin S Widespread and sustained cognitive deficits in alcoholism: a meta-analysis. Addict Biol (2013) 18:203–13 10.1111/j.1369-1600.2011.00418.x 22264351

[36] Ron MA The alcoholic brain: CT scan and psychological findings. Psychol Med Monogr Suppl (1983) 3:1–33 10.1017/S0264180100000345 6573696

[37] Moselhy HF Georgiou G Kahn A Frontal lobe changes in alcoholism: a review of the literature. Alcohol Alcohol (2001) 36:357–68 10.1093/alcalc/36.5.357 11524299

[38] Kril JJ Halliday GM Svoboda MD Cartwright H The cerebral cortex is damaged in chronic alcoholics. Neuroscience (1997) 79:983–98 10.1016/S0306-4522(97)00083-3 9219961

[39] Pfefferbaum A Sullivan EV Mathalon DH Lim KO Frontal lobe volume loss observed with magnetic resonance imaging in older chronic alcoholics. Alcohol Clin Exp Res (1997) 21:521–9 10.1111/j.1530-0277.1997.tb03798.x 9161613

[40] Oscar-Berman M Marinkovic K Alcoholism and the brain: an overview. Alcohol Res Health (2003) 27:125–33 15303622 PMC6668884

[41] Agartz I Momenan R Rawlings RR Kerich MJ Hommer DW Hippocampal volume in patients with alcohol dependence. Arch Gen Psychiatry (1999) 56:356–63 10.1001/archpsyc.56.4.356 10197833

[42] Beresford TP Arciniegas DB Alfers J Clapp L Martin B Du Y Hippocampus volume loss due to chronic heavy drinking. Alcohol Clin Exp Res (2006) 30:1866–70 10.1111/j.1530-0277.2006.00223.x 17067350

[43] Bleich S Sperling W Degner D Graesel E Bleich K Wilhelm J Lack of association between hippocampal volume reduction and first-onset alcohol withdrawal seizure. A volumetric MRI study. Alcohol Alcohol (2003) 38:40–4 10.1093/alcalc/agg017 12554606

[44] Sullivan EV Marsh L Mathalon DH Lim KO Pfefferbaum A Anterior hippocampal volume deficits in nonamnesic, aging chronic alcoholics. Alcohol Clin Exp Res (1995) 19:110–22 10.1111/j.1530-0277.1995.tb01478.x 7771636

[45] Copersino ML Fals-Stewart W Fitzmaurice G Schretlen DJ Sokoloff J Weiss RD Rapid cognitive screening of patients with substance use disorders. Exp Clin Psychopharmacol (2009) 17:337–44 10.1037/a0017260 19803633 PMC3144764

[46] Popovic IM Seric V Demarin V Mild cognitive impairment in symptomatic and asymptomatic cerebrovascular disease. J Neurol Sci (2007) 257:185–93 10.1016/j.jns.2007.01.029 17328916

[47] Noël X Brevers D Bechara A A neurocognitive approach to understanding the neurobiology of addiction. Curr Opin Neurobiol (2013) 23:632–8 10.1016/j.conb.2013.01.018 23395462 PMC3670974

[48] Bechara A Noël X Crone EA Loss of willpower: abnormal neural mechanism of impulse control and decision making in addiction. In: Weirs RW Stacy AW, editors. Handbook of Implicit Cognition and Addiction. Thousand Oaks, CA: SAGE Publishers (2006). p. 215–32

[49] Thush C Wiers RW Ames SL Grenard JL Sussman S Stacy AW Interactions between implicit and explicit cognition and working memory capacity in the prediction of alcohol use in at-risk adolescents. Drug Alcohol Depend (2008) 94:116–24 10.1016/j.drugalcdep.2007.10.019 18155856 PMC3413632

[50] Houben K Havermans RC Nederkoorn C Jansen A Beer à no-go: learning to stop responding to alcohol cues reduces alcohol intake via reduced affective associations rather than increased response inhibition. Addiction (2012) 107:1280–7 10.1111/j.1360-0443.2012.03827.x 22296168

[51] Houben K Nederkoorn C Wiers RW Jansen A Resisting temptation: decreasing alcohol-related affect and drinking behavior by training response inhibition. Drug Alcohol Depend (2011) 116:132–6 10.1016/j.drugalcdep.2010.12.011 21288663

[52] Houben K Wiers RW Jansen A Getting a grip on drinking behavior: training working memory to reduce alcohol abuse. Psychol Sci (2011) 22:968–75 10.1177/0956797611412392 21685380

[53] Houben K Wiers RW Implicitly positive about alcohol? Implicit positive associations predict drinking behavior. Addict Behav (2008) 33:979–86 10.1016/j.addbeh.2008.03.002 18434034

[54] Jajodia A Earleywine M Measuring alcohol expectancies with the implicit association test. Psychol Addict Behav (2003) 17:126–33 10.1037/0893-164X.17.2.126 12814276

[55] Gazdzinski S Durazzo TC Meyerhoff DJ Temporal dynamics and determinants of whole brain tissue volume changes during recovery from alcohol dependence. Drug Alcohol Depend (2005) 78:263–73 10.1016/j.drugalcdep.2004.11.004 15893157

[56] Gazdzinski S Durazzo TC Mon A Yeh P-H Meyerhoff DJ Cerebral white matter recovery in abstinent alcoholics – a multimodality magnetic resonance study. Brain (2010) 133:1043–53 10.1093/brain/awp343 20133395 PMC2850577

[57] Monnig MA Tonigan JS Yeo RA Thoma RJ McCrady BS White matter volume in alcohol use disorders: a meta-analysis. Addict Biol (2013) 18:581–92 10.1111/j.1369-1600.2012.00441.x 22458455 PMC3390447

[58] Bendszus M Weijers HG Wiesbeck G Warmuth-Metz M Bartsch AJ Engels S Sequential MR imaging and proton MR spectroscopy in patients who underwent recent detoxification for chronic alcoholism: correlation with clinical and neuropsychological data. AJNR Am J Neuroradiol (2001) 22:1926–32 11733327 PMC7973857

[59] Agartz I Brag S Franck J Hammarberg A Okugawa G Svinhufvud K MR volumetry during acute alcohol withdrawal and abstinence: a descriptive study. Alcohol Alcohol (2003) 38:71–8 10.1093/alcalc/agg020 12554612

[60] Bartsch AJ Homola G Biller A Smith SM Weijers H-G Wiesbeck GA Manifestations of early brain recovery associated with abstinence from alcoholism. Brain (2007) 130:36–47 10.1093/brain/awl303 17178742

[61] Fein G Torres J Price LJ Di Sclafani V Cognitive performance in long-term abstinent alcoholic individuals. Alcohol Clin Exp Res (2006) 30:1538–44 10.1111/j.1530-0277.2006.00185.x 16930216 PMC1868685

[62] Pitel AL Rivier J Beaunieux H Vabret F Desgranges B Eustache F Changes in the episodic memory and executive functions of abstinent and relapsed alcoholics over a 6-month period. Alcohol Clin Exp Res (2009) 33:490–8 10.1111/j.1530-0277.2008.00859.x 19120052

[63] Mann K Günther A Stetter F Ackermann K Rapid recovery from cognitive deficits in abstinent alcoholics: a controlled test-retest study. Alcohol Alcohol (1999) 34:567–74 10.1093/alcalc/34.4.567 10456585

[64] Manning V Wanigaratne S Best D Hill RG Reed LJ Ball D Changes in neuropsychological functioning during alcohol detoxification. Eur Addict Res (2008) 14:226–33 10.1159/000156479 18810242

[65] Sullivan EV Rosenbloom MJ Lim KO Pfefferbaum A Longitudinal changes in cognition, gait, and balance in abstinent and relapsed alcoholic men: relationships to changes in brain structure. Neuropsychology (2000) 14:178–88 10.1037/0894-4105.14.2.178 10791858

[66] Fama R Pfefferbaum A Sullivan EV Perceptual learning in detoxified alcoholic men: contributions from explicit memory, executive function, and age. Alcohol Clin Exp Res (2004) 28:1657–65 10.1097/01.ALC.0000145690.48510.DA 15547452

[67] Marinkovic K Oscar-Berman M Urban T O’Reilly CE Howard JA Sawyer K Alcoholism and dampened temporal limbic activation to emotional faces. Alcohol Clin Exp Res (2009) 33:1880–92 10.1111/j.1530-0277.2009.01026.x 19673745 PMC3543694

[68] Chanraud S Pitel A-L Müller-Oehring EM Pfefferbaum A Sullivan EV Remapping the brain to compensate for impairment in recovering alcoholics. Cereb Cortex (2013) 23:97–104 10.1093/cercor/bhr381 22275479 PMC3513953

[69] Day E Bentham PW Callaghan R Kuruvilla T George S Thiamine for prevention and treatment of Wernicke-Korsakoff Syndrome in people who abuse alcohol. Cochrane Database Syst Rev (2013) 7:CD004033 10.1002/14651858.CD004033.pub3 23818100 PMC7163251

[70] Morgenstern J Longabaugh R Cognitive-behavioral treatment for alcohol dependence: a review of evidence for its hypothesized mechanisms of action. Addiction (2000) 95:1475–90 10.1046/j.1360-0443.2000.951014753.x 11070524

[71] Premkumar P Fannon D Kuipers E Peters ER Anilkumar APP Simmons A Structural magnetic resonance imaging predictors of responsiveness to cognitive behaviour therapy in psychosis. Schizophr Res (2009) 115:146–55 10.1016/j.schres.2009.08.007 19734016 PMC2845808

[72] Kumari V Peters ER Fannon D Antonova E Premkumar P Anilkumar AP Dorsolateral prefrontal cortex activity predicts responsiveness to cognitive-behavioral therapy in schizophrenia. Biol Psychiatry (2009) 66:594–602 10.1016/j.biopsych.2009.04.036 19560121 PMC2734077

[73] Goldapple K Segal Z Garson C Lau M Bieling P Kennedy S Modulation of cortical-limbic pathways in major depression: treatment-specific effects of cognitive behavior therapy. Arch Gen Psychiatry (2004) 61:34–41 10.1001/archpsyc.61.1.34 14706942

[74] Hunt SA Baker AL Michie PT Kavanagh DJ Neurocognitive profiles of people with comorbid depression and alcohol use: implications for psychological interventions. Addict Behav (2009) 34:878–86 10.1016/j.addbeh.2009.03.036 19398163

[75] Mohlman J Gorman JM The role of executive functioning in CBT: a pilot study with anxious older adults. Behav Res Ther (2005) 43:447–65 10.1016/j.brat.2004.03.007 15701356

[76] Granholm E McQuaid JR Link PC Fish S Patterson T Jeste DV Neuropsychological predictors of functional outcome in Cognitive Behavioral Social Skills Training for older people with schizophrenia. Schizophr Res (2008) 100:133–43 10.1016/j.schres.2007.11.032 18222648 PMC2352154

[77] Allen DN Goldstein G Seaton BE Cognitive rehabilitation of chronic alcohol abusers. Neuropsychol Rev (1997) 7:21–39 10.1007/BF02876971 9243529

[78] Goldstein G Haas GL Shemansky WJ Barnett B Salmon-Cox S Rehabilitation during alcohol detoxication in comorbid neuropsychiatric patients. J Rehabil Res Dev (2005) 42:225–34 10.1682/JRRD.2004.03.0040 15944887

[79] Goldman MS Experience-dependent neuropsychological recovery and the treatment of chronic alcoholism. Neuropsychol Rev (1990) 1:75–101 10.1007/BF01108859 2152526

[80] Le Berre A-P Rauchs G La Joie R Segobin S Mézenge F Boudehent C Readiness to change and brain damage in patients with chronic alcoholism. Psychiatry Res (2013) 213:202–9 10.1016/j.pscychresns.2013.03.009 23838052

[81] Allsop S Saunders B Phillips M The process of relapse in severely dependent male problem drinkers. Addiction (2000) 95:95–106 10.1046/j.1360-0443.2000.9519510.x 10723834

[82] Noël X Sferrazza R Van Der Linden M Paternot J Verhas M Hanak C Contribution of frontal cerebral blood flow measured by (99m)Tc-Bicisate spect and executive function deficits to predicting treatment outcome in alcohol-dependent patients. Alcohol Alcohol (2002) 37:347–54 10.1093/alcalc/37.4.347 12107037

[83] Bates ME Buckman JF Nguyen TT A role for cognitive rehabilitation in increasing the effectiveness of treatment for alcohol use disorders. Neuropsychol Rev (2013) 23:27–47 10.1007/s11065-013-9228-3 23412885 PMC3610413

[84] Prochaska JO DiClemente CC Stages and processes of self-change of smoking: toward an integrative model of change. J Consult Clin Psychol (1983) 51:390–5 10.1037/0022-006X.51.3.390 6863699

[85] Rupp CI Kemmler G Kurz M Hinterhuber H Fleischhacker WW Cognitive remediation therapy during treatment for alcohol dependence. J Stud Alcohol Drugs (2012) 73:625–34 22630801 10.15288/jsad.2012.73.625

[86] Grenard JL Ames SL Wiers RW Thush C Sussman S Stacy AW Working memory capacity moderates the predictive effects of drug-related associations on substance use. Psychol Addict Behav (2008) 22:426–32 10.1037/0893-164X.22.3.426 18778136 PMC3119708

[87] Houben K Wiers RW Response inhibition moderates the relationship between implicit associations and drinking behavior. Alcohol Clin Exp Res (2009) 33:626–33 10.1111/j.1530-0277.2008.00877.x 19183132

[88] Seron X Van der Linden M Objectifs et stratégies de la revalidation neuropsychologique. In: Seron X Van der Linden M, editors. Traité de Neuropsychologie Clinique: Tome 2. Marseille: Solal (2000). p. 9–16

